# Editorial: Using Cells in Epidemiological Studies to Characterize Individual Response to Environmental Hazards

**DOI:** 10.3389/fpubh.2019.00284

**Published:** 2019-10-02

**Authors:** Dora Il'yasova, Alexander V. Kinev

**Affiliations:** ^1^Department of Population Health Sciences, School of Public Health, Georgia State University, Atlanta, GA, United States; ^2^Creative Scientist Inc., Research Triangle Park, NC, United States

**Keywords:** epidemiology, susceptibility, low-dose exposure, cell-based assays, individual variability, risk stratification

The current advances in whole genome sequencing provided mounting evidence about human genetic variability (1). Genetic variability largely contributes to the differences in individual responses to chemicals; however, translation of individual or population-wide genomic data into prediction of human phenotypical responses to external factors remains problematic (2–4). In this regard, studying responses of cells derived from individuals represent an opportunity to gain insight into both individual susceptibility and population-wide variability to the known or potential environmental toxicants. In many aspects, studying of *in vitro* cellular responses to xenobiotics is similar to the *in vivo* challenge tests, such as oral glucose tolerance test (determining predisposition to developing type 2 diabetes) (5) or exercise test (used for the diagnosis of obstructive coronary artery disease) (6). The main purpose of the challenge testing is to rank individuals as more or less susceptible to a challenge. Cell-based testing with exposure to xenobiotics follows the same concept, providing information for risk stratification in epidemiological studies. Donor-specific cells can be used as a proxy of individuals to assess susceptibility to external factors. Ranking of individuals by cellular responses translates genetic variability into biologically meaningful endpoints, such as changes in cell cycle progression, death, or differentiation. Furthermore, cell-based assays may examine metabolic transformations as well as the role of cellular organelles, such as mitochondria, in variability of donor-specific responses. Thus, cellular responses can potentially portray individual sensitivity or resilience to environmental toxicants and ultimately serve as a foundation for the analysis of population-wide variability in phenotypical responses to xenobiotics. These research areas have explored how and whether human variability in response to possible exposures can be accessed using human cells. Currently, human cells are used in cell-based toxicology—a fast growing field that has both advantages (low cost and human relevance) and deficiencies (availability of human cells and lack of standardization). Building upon the methods of cellular toxicology, cellular epidemiology can advance assessment of human susceptibility (or resilience) to environmental pressures. The presented collection of five manuscripts provides an interdisciplinary perspective on the potential of moving epidemiology to a new level in the characterization of individual susceptibility to environmental exposures.

The study by Filonov et al. aims to answer the question whether donor-specific cells could be used as a proxy for an individual in characterizing cellular response to environmental toxicants. While individual responses to toxicants have been previously characterized using transformed lymphoblastoid cells (7), the main advantage of this study lies in using primary human cells, specifically, endothelial colony-forming (progenitor) cells (ECFCs). In this study, the authors suggest that response of neonatal ECFCs to toxicants may functionally be linked to the toxicity in fetus as vasculature presents the earliest integral part of all tissues (8). Based on this premise, four known toxic chemicals and their four relatively non-toxic counterparts were tested in eight ECFC clones representing 2 male and 2 female donors (2 clones per donor). This study unequivocally demonstrated that all ECFC clones correctly distinguished between toxic and less toxic chemicals, validating this cellular model as toxicologically relevant. In particular, this initial evaluation showed that in response to menadione, clones from the same donor exhibited similar responses with a larger variability between the donors. This finding suggests that donor-specific cells may present an adequate model for characterizing individual differences in response to environmental exposures.

In the second article, the authors Il'yasova et al. expressed another idea related to donor-specific cells. The cells present an opportunity to study individual differences in mitochondrial health through the direct assessment of mitochondrial function. Such measurements known as a “stress test” have been conducted for decades in isolated mitochondria; and the functional value of the results were well-established in bioenergetics (9). The recent progress in cell biology, i.e., isolation of rare stem and progenitor cells from human tissues in combination with the development of high-resolution respirometry technology, provides new opportunities to evaluate mitochondrial health in donor-specific cells. The authors specifically discuss application of this approach to studying perinatal environmental toxicology. Umbilical cord blood presents a rich source of stem and progenitor cells, providing a unique opportunity to procure fetal/neonatal biological material in a non-invasive manner. As mitochondria are thought to be very sensitive to environmental toxicants (10), donor-specific cells isolated from cord blood may serve to study mitochondrial health in newborns, the population that is both very vulnerable and otherwise inaccessible for procurement of biological material.

The study by Forsythe et al. explores the use of 3D organoids, one of the most advanced *in vitro* cellular platforms, for toxicological research. 3D organoids are self-organizing organ-specific cellular macrostructures that resemble the *in vivo* tissue both structurally and physiologically (11). The advantage of this model lies in capturing complex cell-cell and cell-matrix interactions, which are not present in traditional 2D cell culture models. Whereas, organoids are already being used in drug discovery and analysis, the application of this model for the screening of potential environmental toxicants has been limited. The authors describe the response of liver and cardiac organoids to four established environmental pollutants that have a considerable public attention—three toxic metals (lead, mercury, thallium) and a pesticide (glyphosphate). By testing different aspects of cell metabolism, the authors demonstrated that these toxicants reduced ATP content and inhibited cell viability in liver and cardiac organoids. The authors show that functional heartbeat assays in cardiac organoids can produce toxicity data much faster and more cost-efficiently as compared to animal studies. Together, the findings suggest that 3D organoids can serve for tissue-specific evaluation of toxicity to screen newly developed chemical products.

In a review article, Truskey puts into perspective utilization of human microphysiological systems, including organoids, in toxicological testing. The review highlights advantages and limitations of microphysiological systems. Microphysiological systems provide an opportunity to evaluate context-dependent impact of environmental pollutants. Whereas salient theoretically, this approach currently faces technical limitations, such as isolation of primary cells, their differentiation, and scaling-up the through-put of toxicity assays. However, the author envisions how linking organoids and micromachine devices can produce more accurate modeling of human organ system, replication multiple processes involved in toxicological effects—absorption, disposition, metabolism, and excretion. Extrapolation of such microphysiological systems to studying human variability in response to toxicants seems to be far-fetched today; however, the technological progress in cell-based systems may bring this idea to the reality.

Finally, Zhang et al. discuss how data from human cells and microphysiological systems can be incorporated into the existing knowledge from animal models with the ultimate goal of chemical safety assessment. This review highlights the necessity of moving risk assessment from animal models to human cells, because variability of human responses to xenobiotics cannot not be predicted by animal testing (12–15). The authors discuss computational toxicology as bridging the gap between *in vitro* models and human populations.

The presented review of the topic obviously cannot be comprehensive. Cellular toxicology is growing at a fast pace due to (a) technical improvement in primary cell isolation and culture techniques; (b) the development of methods of making cellular organoids, in particular, an ability of growing different cell types in three-dimensional space; (c) the introduction of donor-derived induced pluripotent cells including disease-specific cells; and (d) the progress of “organ-on-chip” concept to working prototypes. Many questions will be resolved or brought forth in future studies. Since cells specialize within the body, it is important to determine which cell types can best characterize the susceptibility (or resilience) of a person. Such studies can examine correlations between responses of different cell types isolated from the same individual. Moreover, a study that examines correlations between cellular and total body responses to a challenge would be very informative. For example, such studies can be conducted in groups of patients undergoing chemotherapy, as experimental exposure of humans to chemicals would be unethical. Also, it is important to examine how accumulation of somatic mutations and epigenetic changes with aging changes cellular responses to chemicals. Together with advancement in computational toxicology the presented manuscripts describe conceptual and methodological approaches to studying human variability in response to chemicals. All these developments applied to human populations will create a continuum between toxicology and epidemiology.

## Author Contributions

DI provided initial draft. AK discussed and provided edits.

### Conflict of Interest

AK is a founder and executive director of Creative Scientist, Inc. The authors declare that the research was conducted in the absence of any commercial or financial relationships that could be construed as a potential conflict of interest.
